# Potential Applications of Essential Oils for Environmental Sanitization and Antimicrobial Treatment of Intensive Livestock Infections

**DOI:** 10.3390/microorganisms10040822

**Published:** 2022-04-15

**Authors:** Melinda Mariotti, Giulia Lombardini, Silvia Rizzo, Donatella Scarafile, Monica Modesto, Eleonora Truzzi, Stefania Benvenuti, Alberto Elmi, Martina Bertocchi, Laura Fiorentini, Lorenzo Gambi, Maurizio Scozzoli, Paola Mattarelli

**Affiliations:** 1 Dipartimento di Scienze Biotecnologiche di Base, Cliniche Intensivologiche e Perioperatorie, Università Cattolica del Sacro Cuore, Largo A. Gemelli 8, 00168 Rome, Italy; giulia.lombardini@unicatt.it (G.L.); silvia.rizzo01@icatt.it (S.R.); 2 Dipartimento di Scienze e Tecnologie Agro-Alimentari, Università di Bologna, Viale G. Fanin 42, 40127 Bologna, Italy; donatella.scarafile2@unibo.it (D.S.); monica.modesto@unibo.it (M.M.); paola.mattarelli@unibo.it (P.M.); 3 Dipartimento di Scienze della Vita, Università di Modena e Reggio Emilia, Via G. Campi 103, 41125 Modena, Italy; eleonora.truzzi@unimore.it (E.T.); stefania.benvenuti@unimore.it (S.B.); 4 Dipartimento di Scienze Mediche Veterinarie, Università di Bologna, Via Tolara di Sopra 50, Ozzano dell’Emilia, 40064 Bologna, Italy; alberto.elmi2@unibo.it (A.E.); martina.bertocchi3@unibo.it (M.B.); 5 Istituto Zooprofilattico Sperimentale della Lombardia e dell’Emilia Romagna (IZSLER)—Sede Territoriale di Forlì, Via Don Eugenio Servadei 3E/3F, 47122 Forlì, Italy; laura.fiorentini@izsler.it (L.F.); lorenzo.gambi@izsler.it (L.G.); 6 Società Italiana per la Ricerca sugli Oli Essenziali (SIROE), Viale Regina Elena 299, 00161 Rome, Italy; mscozzoli@gmail.com

**Keywords:** *Salmonella* spp., *Clostridium* spp., *Escherichia coli*, poultry, swine

## Abstract

The extensive use of antibiotics has contributed to the current antibiotic resistance crisis. Livestock infections of *Salmonella* spp, *Clostridium* spp. and *E. coli* antimicrobial-resistant bacteria represent a public threat to human and animal health. To reduce the incidence of these zoonoses, essential oils (EOs) could be effective antibiotic alternatives. This study aims at identifying EOs safe for use, effective both in complementary therapy and in the environmental sanitization of intensive farming. Natural products were chemo-characterized by gas chromatography. Three *S.* Typhimurium, three *C. perfringens* and four *E. coli* strains isolated from poultry and swine farms were used to assess the antimicrobial properties of nine EOs and a modified GR-OLI (mGR-OLI). The toxicity of the most effective ones (*Cinnamomum zeylanicum*, Cz; *Origanum vulgare*, Ov) was also evaluated on porcine spermatozoa and *Galleria mellonella* larvae. Cz, Ov and mGR-OLI showed the strongest antimicrobial activity; their volatile components were also able to significantly inhibit the growth of tested strains. *In vitro*, Ov toxicity was slightly lower than Cz, while it showed no toxicity on *G. mellonella* larvae. In conclusion, the study confirms the importance of evaluating natural products to consolidate the idea of safe EO applications in reducing and preventing intensive livestock infections.

## 1. Introduction

The demand for animal-origin food for human consumption is rapidly increasing, especially in low-income countries, representing one of the main challenges of world livestock. This growing trend is changing animal production practices, and local farms are promptly being replaced by market-oriented, large-scale, intensive livestock systems [1]. To meet the global population’s food requirements, antibiotics have become an essential component in animal husbandry, being extensively used not only for therapeutic aims but also for preventing disease as prophylactic drugs and as growth promoters [2,3]. Despite the benefits of treating animal diseases using antibiotics, their excessive use has generated a higher selective pressure that has contributed to the current antibiotic resistance emergence. In 2014, the World Health Organization (WHO) stated that the bacteria antibiotic resistance had become an imminent public health crisis with a significant impact worldwide [4].

Today, antimicrobial-resistant bacteria (ARBs) infections are becoming more frequent every day [5], and it has been estimated that these infections will cause 10 million deaths per year by 2050 [6]. ARBs infections of food animals are particularly concerning; they implicate veterinary care costs, increased mortality, reduced populations and productivity [7]. Sick animals can infect other members of their flock directly or by contaminating environmental sources, such as feed, water, soil and vegetables, especially in intensive livestock where space per animal is limited [8]. Furthermore, bacteria could also spread from carcass to carcass at any stage of animal processing in slaughterhouses [9,10]. Since the outbreaks of zoonotic diseases in humans are associated with direct or indirect contact with an infected animal, animal’s environment or, more commonly, by consumption of contaminated animal origin food, livestock infections have an enormous impact on public health as well, and huge efforts are made to control them [2,11].

According to the 2019 report on zoonosis by the European Food Safety Authority (EFSA) and the European Centre for Disease Prevention and Control (ECDC), salmonellosis was the second major cause of foodborne enteric disease [12], and it is one of the four key global causes of diarrheal diseases [13].

*Salmonella* spp. are Gram-negative non-spore-forming bacteria that are members of the *Enterobacteriaceae* family [14] that pose a great threat to the food industry, with chickens and pigs as *Salmonella*’s main vehicles. These bacteria have a broad host range, causing, from asymptomatic to severe, infections in animals, as well as typhoid fever or acute diarrhea and other symptoms in humans [15].

In intensive livestock, *Clostridium* spp. is an important problem too. It is a genus of anaerobic, Gram-positive, spore-forming bacteria of the *Clostridiaceae* family [16]; most of its species are members of the gut microbiota in humans and animals or inhabitants of the soil. Only a few strains are pathogenic that can contribute to several diseases both in animals and humans, such as neurotoxic and enteric ones [17,18]. Chickens are severely affected by clostridial necrotic enteritis (NE) [19], a potentially fatal gastrointestinal condition, and are known as a vehicle for enteric human *Clostridium* spp. disease [20,21].

*Escherichia coli* is a facultative anaerobic Gram-negative bacterium belonging to the *Enterobacteriaceae* family; commonly, it resides in mammals and humans’ intestines as commensal, where it coexists with its hosts with mutual benefit [22,23]. *E. coli* is also an important pathogen responsible for enteritis, urinary tract infections, peritonitis and meningitis in humans, and it is an important cause of several diseases in swine where it is mainly associated with diarrhea [24,25].

The spread of ARBs is accelerated by long-term antibiotic use at lower doses than the treatment ones. To minimize this global issue and, at the same time, to ensure the safety of animal-origin food, the World Organization for Animal Health (OIE) recommended a responsible and rational use of antimicrobial drugs in animals [26]. Several countries have already taken action to decrease the level of antimicrobial drugs used in animal farms: in 2006, the European Union (EU), for example, banned their use as growth promoters in animal feeds [27]. Therefore, this has pressured the farm industries to look for efficient antibiotic substitutes along with improvement of animal welfare and biosecurity measures in order to reduce the incidence of the most important zoonosis in intensive poultry and swine livestock. Among the extensively studied antibiotic alternatives, there are condensed tannins [28], phages [29,30], vaccines [31], probiotics [32], acidifiers [33], oligosaccharides [34], plant extracts [35] and, last but not least, essential oils (EOs) [36].

EOs are highly concentrated, aromatic and volatile substances of plant origin, including flowers, roots, bark, leaves, seeds, fruits and wood formed in the cytoplasm and normally present in the form of droplets between cells. As defined by official Pharmacopoeias, EOs are considered as complex odorous products obtained by steam distillation or by hydro-distillation, or by the dry distillation of a plant or some of its parts or, in the case of OEs obtained from *Citrus* spp., through appropriate mechanical cold processes [37]. Whereas the International Organization for Standardization (ISO) established both criteria of concept and quality criteria, which are worldwide guidelines for EOs [38].

Many studies have examined EOs’ *in vitro* and *in vivo* efficiency. Some of them have evaluated the effects of EOs on alive animals by supplementing animal feed with EOs [39,40] or by misting them in animals’ rooms to reduce bacterial contamination and improve hygiene standards on farms [41]. Other studies were conducted on bacteria strains isolated from farm animals [42,43,44], on reference strains [45,46], on seminal swine doses to reduce bacterial load [47] and on animal origin food [48,49] to decrease bacterial contaminations and preserve it, thus enhancing the shelf-life of the products destined for human consumption.

The aim of this study is to identify EOs that are contextually safe for use and effective both in the fight against antibiotic resistance, as in the alternative therapy to synthetic antibiotic drugs, and in the environmental sanitization of intensive farming.

## 2. Materials and Methods

### 2.1. Natural Substances and Reagents

Nine pure essential oils and a blend were used for the study. In particular, *Eucaliptus globus*, *Origanum vulgare* (Ov), *Melaleuca alternifolia*, *Lavandula angustifolia*, *Melaleuca leucadendron*, *Citrus limon* (Cl), *Cinnamonum zeylanicum* (Cz) from leaves, *Lavandula × hybrida*, *Mentha piperita* and a modified mix (formula unknown) of GR-OLI (a confidential solution under patent processing, containing nine EOs *E. globulus*, *Satureja hortensis*, *Citrus aurantium var. dulcis*, *Thymus vulgaris*, *M. alternifolia*, *C. limon*, *L. × hybrida*, *Melaleuca leucadendron* and *Thymus capitatus*) dispersed in the surfactant Glyceryl polyethyleneglycol ricinoleate (cod. E484) (mGR-OLI) were provided by APA-CT (Forlì, Italy).

*n*-hexane (Hex) and C_8_-C_40_ n-Alkanes Calibration Standard were of analytical grade from Merck Life Science (Milan, Italy).

### 2.2. GC-MS Analysis

Analyses were performed on a 7890A gas chromatograph coupled with a 5975C network-mass spectrometer (GC-MS) (Agilent Technologies, Milan, Italy). Compounds were separated on an Agilent Technologies HP-5 MS cross-linked poly-5% diphenyl–95% dimethyl polysiloxane (30 m × 0.25 mm i.d., 0.25 μm film thickness) capillary column according to a gradient temperature program to obtain better separation of the peaks and to allow the complete elution of all components. The column temperature was initially set at 45 °C, then increased at a rate of 2 °C/min up to 100 °C, then raised to 250 °C at a rate of 5 °C/min, and, finally, held for 5 min. The injection volume was 0.1 μL, with a split ratio 1:20. Helium was used as the carrier gas at a flow rate of 0.7 mL/min. The injector, transfer line and ion-source temperature were 250, 280, and 230 °C, respectively. MS detection was performed with electron ionization (EI) at 70 eV, operating in the full-scan acquisition mode in the *m*/*z* range 40–400. The EOs were diluted 1:20 (*v*/*v*) with *n*-hexane before GC-MS analysis.

### 2.3. GC-FID Analysis

The chromatographic characterization of EOs was performed on a 7820 gas chromatograph (Agilent Technologies, Milan, Italy) with a flame ionization detector (FID). EOs and the mixture of aliphatic hydrocarbons (C_8_–C_40_) were diluted 1:20 (*v*/*v*) with Hex before GC-FID analysis. Helium was used as the carrier gas at a flow rate of 1 mL/min with a pressure of 2.5 bar at the column head. The injector and detector temperatures were set at 250 and 300 °C, respectively. EO components were separated on an Agilent Technologies HP-5 cross-linked poly-5% diphenyl–95% dimethylsiloxane (30 m × 0.32 mm i.d., 0.25 mm film thickness) capillary column. The column temperature was initially set at 45 °C, then increased at a rate of 2 °C/min up to 100 °C, then raised to 250 °C at a rate of 5 °C/min, and, finally, maintained for 5 min. The injection volume was 1 μL, with a split ratio 1:20.

Compounds were identified by comparing the retention times of the chromatographic peaks with those of authentic reference standards run under the same conditions and by comparing the linear retention indices (*LRI*s) relative to C_8_–C_40_ *n*-alkanes obtained on the HP-5 column under the above-mentioned conditions in the literature [50]. Peak enrichment by co-injection with authentic reference compounds was also carried out. A comparison of the MS-fragmentation pattern of the target analytes with those of pure components was performed by using the National Institute of Standards and Technology (NIST version 2.0d, 2005) mass-spectral database.

The relative percentage amount of individual components was expressed as the percent peak area relative to the total peak area obtained by the GC/FID analysis. Semi-quantitative data were acquired from the mean of two analyses. The percentages of each compound are expressed as the mean ± standard deviation (SD) of the three replicates for each kind of treatment.

The data acquisition and processing were performed using the OpenLab CDS C.01.04 (Agilent Technologies, Santa Clara, CA, USA) software.

### 2.4. Bacterial Strains and Growth Media

To study the efficacy of the natural products, EOs were tested on 3 isolates of *Enterobacteriaceae Salmonella* Typhimurium, 3 strains of *Clostridium perfringens*, and 4 strains of *E. coli* by Istituto Zooprofilattico of Forlì (Italy) (Table 1). Both *S*. Typhimurium and *E. coli* strains were incubated at 37 ± 1 °C with 5% CO_2_ overnight, and *C. perfringens* at 37 ± 1 °C in anaerobic conditions for 48 h.

### 2.5. Antimicrobial Susceptibility Testing

The antimicrobial susceptibility of the tested bacterial strains was performed according to the Kirby-Bauer disk diffusion method. For the *Enterobacteriaceae* isolates, the following panel of antimicrobials was tested: Nalidixic acid (NA), Aminosidine (AN), Amoxicillin/Clavulanic acid association (AMC), Ampicillin (AMP), Apramycin (APR), Cefazolin (KZ), Colistin (CT), Enrofloxacin (ENR), Florfenicol (FFC), Gentamicin (CN), Kanamycin (K), tetracycline (TE), Tilmicosin (TIL) and Sulfamethoxazole/Trimethoprim association (SXT). Different antimicrobial molecules are needed for clostridial inhibition; thus the following panel was used for the tested *Clostridium perfringens* isolates: Amoxicillin (AX), Bacitracin (B), Doxycycline (DXT), Erythromycin (E), Lincomycin (MY), Penicillin (PV), Spiramycin (SP), Tetracycline (TE), Tiamulin (T) and Tylosin (TY). The zone of inhibition interpretation was performed in agreement with the guidelines by Centro Nazionale di Referenza per l’Antibiotico Resistenza (CRAB) and the Clinical and Laboratory Standards Institute (CLSI) [51,52,53,54,55,56,57].

### 2.6. Preparation of EOs

EOs were prepared as oil-in-water emulsions for the evaluation of their antimicrobial properties. Each EO emulsion consisted of 4% EO (*v*/*v*) 2% Tween 80 (*v*/*v*) (Sigma-Aldrich, Saint Louis, MO, USA) in Müller–Hinton culture medium broth (Mueller–Hinton Broth Oxoid Ltd., CM0405, Wade Road, Basingstoke, Hants, RG24 8 PW, UK and Supplemented Brucella Broth Thermo Scientific T345-330155, Lenexa, KS, USA), the only exception was Cz EO was mixed (1% *v*/*v*) together with 0.5% *v*/*v* of Tween 80 (*v*/*v*) in culture medium broth. The emulsions were vortexed for 1 min before use and were used for microbroth dilution, micro-atmosphere diffusion assay and *in vivo* toxicity on *Galleria mellonella* larvae. No preservatives or other substances were added to the mixture.

### 2.7. Broth Microdilution Susceptibility Testing

Broth microdilution (BMD) susceptibility tests were performed according to the European Committee on Antimicrobial Susceptibility Testing (EUCAST) international guidelines [58]. Sensitrite Supplemented Brucella Broth (Thermo Scientific, Lenexa, KS, USA) and Muller–Hilton broth (Oxoid, Basingstoke, Hampshire, UK) were used, respectively, against *C. perfringens* and both *S*. Typhimurium and *E. coli* to test the antimicrobial activity of the Mix and the nine EOs. All BMD tests were performed on a 96-well plate and serial dilutions between 2% *v*/*v* equal to 40 µL of EOs content/mL (0.5% *v*/*v* for Cz) and 0.015% *v*/*v* equal to 0.15 µL of EOs content/mL (Cz 0.003% *v*/*v*) were tested.

A suspension 0.5 McFarland was adjusted in order to have 5 × 10^5^ CFU/mL of bacterial suspension in each well. Plates of *S.* Typhimurium and *E. coli* were incubated overnight at 37 °C for 24 h, while those of *C. perfringens* were kept at 37 °C for 48 h in anaerobic conditions. After the incubation period, the Minimum Inhibitory Concentration (MIC) values were determined. MIC is defined as the lowest concentration of EOs corresponding to the complete inhibition of bacterial growth. To evaluate the Minimum Bactericidal Concentration (MBC), 10 µL of the content of each well was seeded on Difco Mueller–Hinton-Agar (Becton Dickson and Company, Sparks, MD, USA) and Schaedler Agar with Vitamin K1 and 5% Sheep Blood (Becton Dickson and Company, Sparks, MD, USA), which were incubated at 37 °C for 24 and 48 h, respectively. MBC is defined as the lowest concentration of EO corresponding to the absence of bacterial growth.

### 2.8. Micro-Atmosphere Diffusion Assay

To evaluate the antimicrobial activity of volatile compounds of the natural products with the best antimicrobial activity (Cz, Ov and mGR-OLI), the micro-atmosphere diffusion assay was performed. The EO that had shown the lowest antimicrobial activity (Cl), was also tested. Kirby–Bauer micro-disks were applied on the lid of the MH agar Petri dish and soaked with 10 µL of Cl, Cz, Ov EOs or the mGR-OLI. A total of 100 µL of 1 × 10^6^ CFU/mL suspension of *S*. Typhimurium or *E. coli* were inoculated on the agar. Parafilm (Parafilm®, Bemis Company, Inc., Neenah, WI, USA) was used to hermetically seal Petri dishes to prevent EO vapor dispersion. The samples were incubated for 24 h at 37 °C. At the end of the incubation time, the inhibition diameter was measured with a ruler. The positive control was performed without a Kirby–Bauer micro-disk; each experiment was performed in triplicate.

### 2.9. Effect of EOs on Porcine Spermatozoa

Cz and Ov EOs were tested on porcine spermatozoa, as previously reported [59], to preliminary assess overall cytotoxicity. Upon ejaculate collection, the sperm-rich fraction (SFR) was immediately diluted 1:1 *v*/*v* with an in-house prepared swine fertilization medium (SFM) without any antibiotic [60]. To assess overall quality, each SFR was tested for spermatozoa concentration using a Thoma hemocytometer chamber, viability (V) by eosin-nigrosine staining and total motility (TotM) by computer-assisted sperm analysis (CASA; Hamilton Thorne CEROS II; Animal Motility II, Software Version 1.9, Beverly, MA, USA). Only SFR with V > 85% and TotM > 80% were used for the experimental protocol.

Each essential oil was tested in triplicate. The experimental doses were prepared by suspending a fixed number of spermatozoa (15 × 10^7^ spz) in 5 mL of SFM extender (final concentration = 3 × 10^7^ spz/mL) with 3 different concentrations (1, 0.5 and 0.1 mg/mL) of EOs previously added with emulsifiers (DMSO 0.5%, Tween 80 0.02%). For each experiment, control samples were realized by only adding the emulsifiers. After preparation, the experimental doses were stored for 120 h in a refrigerated bath set at 16 °C (±1 °C); at 3 and 120 h, samples were evaluated for the key morpho-functional parameters following previously published protocols: viability by eosin-nigrosin staining, acrosome status by modified Coomassie Blue staining and objective motility by CASA [61].

### 2.10. Toxicity of EOs in Galleria mellonella Larvae

*G. mellonella* larvae were used to test the toxicity of EOs with greater antimicrobial effectiveness (Cz, Ov). Each EO was tested at the highest MIC (0.25% *v*/*v* for Cz; 1% *v*/*v* for Ov) and at 2-4-8 MIC. A mixture of EOs, sterile water and 1% Tween 80 was injected into the treated group larvae, and only sterile water and Tween 80 were used for the control group. The larvae were placed in Petri dishes, incubated in the dark for 72 h at 30 °C and the viability was recorded daily. Larvae were reared on a diet supplemented with milk flour (Dieterba, Heinz Italy, Ozzano Taro PR, Italy) and honey (Mielizia, Monterenzio BO Italy) at 30 °C. Ten larvae (300–450 mg each) were selected randomly for each group of the experiment. Any larvae with dark pigmentations of the cuticle were excluded. A total of 10 µL of each natural product were injected into the hemocoel through the last left proleg (Insumed 30 G Pic, Casnate con Bernate CO, Italy, syringe volume 0.5 mL, needle size 30 G) [62]. Prior to the administration of EOs, the pricked area was decontaminated with 70% ethanol. The experiment was performed at least in duplicate.

### 2.11. Statistical Analysis

The statistical analysis was performed using the software GraphPad Prism v.8 (GraphPad Software Inc., San Diego, CA, USA). To evaluate differences between the control doses and the others containing EOs, one-way ANOVA at each time point was performed with the significance level set at 0.05. *Post hoc* analyses were performed by means of Dunnett’s test (*p* < 0.05) to assess the differences between the controls and the treatments.

## 3. Results

### 3.1. Chemical Composition of the EOs

The main chemical composition of essential oils considered in the study is displayed in Table 2. The essential oils of lavender showed a similar chemical composition, mainly represented by the high concentration of the alcoholic monoterpene linalool and its ester linalyl acetate. According to the literature, the main differences between *L. × intermedia* and *L. angustifolia* EOs are especially related to the different ratios of camphor, 1,8-cineole, borneol and cis ocimene [63,64]. The EOs belonging to the *Melaleuca* genus exhibited an extremely different composition. Specifically, *M. alternifolia* EO displayed a high content of the isomeric monocyclic hydrocarbons α and γ-terpinene and their oxygenated derivatives terpine-4-ol and α-terpineol. On the contrary, *M. leucadendron* EO was characterized by the ether 1,8-cineole and the alcoholic monoterpene p-cymen-8-ol, which represented 82.86% of the total composition. The latter EO showed a similar chemical profile to *E. globulus* EO, where 91.44% of the whole composition was referred to as 1,8-cineole abundance. *Origanum vulgare* EO was classified as a carvacrol chemotype, being rich in the aromatic hydrocarbon p-cymene and its oxygenated derivative carvacrol [65]. The EO derived from the leaves of *Cinnamonum zeylanicum* (also called *C. verum*) displayed a high abundance of the phenolic derivative eugenol (70.43%), followed by neryl acetate (5.84%), β-caryophyllene (4.83%) and linalool (4.27%). The chemical composition of *Citrus limon* EO was almost completely characterized by monocyclic hydrocarbons, namely limonene, α-/β-pinene and γ-terpinene, which accounted for 93.32% of the total composition. Differently, *Mentha piperita* EO exhibited high ratios of oxygenated monoterpenes belonging to different chemical classes, such as alcohols (menthol and neomenthol), ketones (methone), esters (menthyl acetate) and ethers (1,8-cineole). In particular, these monoterpenes were all derivatives of the p-menthane skeleton except for 1,8-cineole. Finally, the mGR-OLI displayed an overall composition that reflected the compositions of the original EOs. Specifically, the most abundant monoterpenes were the hydrocarbons p-cymene, limonene and γ-terpinene, the alcohols linalool, terpinene-4-ol, α-terpineol, geraniol, thymol and carvacrol, the ether 1,8-cineole, and the aldehyde trans-cinnamaldehyde. The whole chemical composition of essential oils is reported in Supplementary Materials (Table S1).

### 3.2. Antimicrobial Susceptibility Testing 

Table 3 shows the sensitivity of all strains of *S.* Typhimurium, *C. perfringens* and *E. coli* against antibiotics commonly used in veterinary medicine. The *S.* Typhimurium strains were all sensitive to nalidixic acid, aminosidine, apramycin, colistin sulfate, enrofloxacin, florfenicol and kanamycin. ST2 and ST3 strains were both resistant to ampicillin and tilmicosin and both susceptible to increased exposure sensitivity to amoxicillin/clavulanic acid and cefazolin; only the ST2 strain was resistant to gentamicin and tetracycline, whereas only ST3 to sulfamethoxazole/trimethoprim. ST1 was sensitive to all antibiotics tested on *Salmonella* strains.

*C. perfringens* strains were all sensitive to bacitracin, spiramycin, tiamulin and tylosin. All strains were resistant to lincomycin, and Cp5 was resistant to tetracycline, amoxicillin, doxycycline, erythromycin and penicillin as well.

All *E. coli* strains showed multi-resistance phenotypes with the exception of strain Ec10, resistant only to tilmicosin and susceptible to increased exposure sensitivity to ampicillin. In particular, Ec7, Ec8 and Ec9 strains were resistant to aminosidine, ampicillin, florfenicol, gentamicin, kanamycin, tetracycline, tilmicosin, and sulfamethoxazole/trimethoprim; Ec8 and Ec9 were both resistant to amoxicillin/clavulanic acid and cefazolin. Among all the strains tested against these antibiotics, Ec8 was the only one resistant to nalidixic acid and enrofloxacin, while Ec9 to apramycin and colistin sulfate.

### 3.3. Broth Microdilution Susceptibility Testing

The *in vitro* sensitivity of *S.* Typhimurium, *C. perfringens* and *E. coli* strains *vs*. *E. globus*, *O. vulgare*, *M. alternifolia*, *L. angustifolia*, *M. leucadendron*, *C. limon*, *C. zeylanicum*, *L. × hybrida*, *M. piperita* and mGR-OLI was investigated through the broth microdilution susceptibility test. As shown in Table 4, the Ov, Cz and the mGR-OLI showed the strongest antimicrobial activity for all strains, with an inhibitory concentration of 90% of strains (IC90) equal to 0.25%, 0.13% and 0.33% respectively, and the minimal inhibitory concentration (MIC) values ranging from 0.06% to 0.38% *v*/*v* for Oz, 0.06% to 0.31% *v*/*v* for Cz and 0.08% to 0.38% *v*/*v* for mGR-OLI. Cl and *M. piperita* had the weakest antimicrobial activity with IC90 >2% and MIC values ranging from 1% to >2% *v*/*v* for the former and from 0.25% to >2% *v*/*v* for the latter.

Ov, Cz and mGR-OLI had the strongest bactericidal activity, with a respective cytocidal concentration of the 90% of strains (CC90) equal to 1%, 0.5% and 0.67%. Minimal bactericidal concentrations (MBC) values ranged in 0.17%–1.17% *v*/*v* for Ov, 0.03%– >0.5% *v*/*v* for Cz and 0.13–1.17% *v*/*v* for mGR-OLI. *E. globus*, *M. leucadendron*, Cl and *M. piperita* showed the lowest bactericidal activity with CC90 >2% (Table 5).

### 3.4. Micro-Atmosphere Diffusion Assay

The antimicrobial activity of natural products’ volatile compounds with the strongest antimicrobial activity (Cz, Ov and mGR-OLI) was evaluated through the micro-atmosphere diffusion assay. The EO that had shown the lowest antimicrobial activity (Cl), was also tested as the negative control.

**Figure 1:**
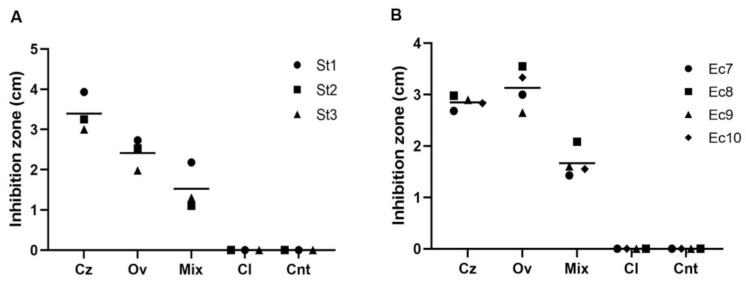
Micro-atmosphere diffusion assay: (**A**). Plot shows inhibition zones of *Salmonella* strains; (**B**). Plot shows inhibition zones of *E. coli* strains.

Figure 1 indicates that Cz, Ov and the mGR-OLI showed significant statistical activity if compared to control values (*p* < 0.00001) at inhibiting all *S.* Typhimurium and *E. coli* strains. In particular, Cz EO turned out to be more efficient against *S.* Typhimurium strains, while Ov gave the highest inhibition zones against *E. coli* strains. As expected, the volatile compounds of Cl EO had no antimicrobial activity against any strains (*p* > 0.05). Raw data are reported in Supplementary Material (Table S2).

### 3.5. Effect of EOs on Porcine Spermatozoa

**Figure 2:**
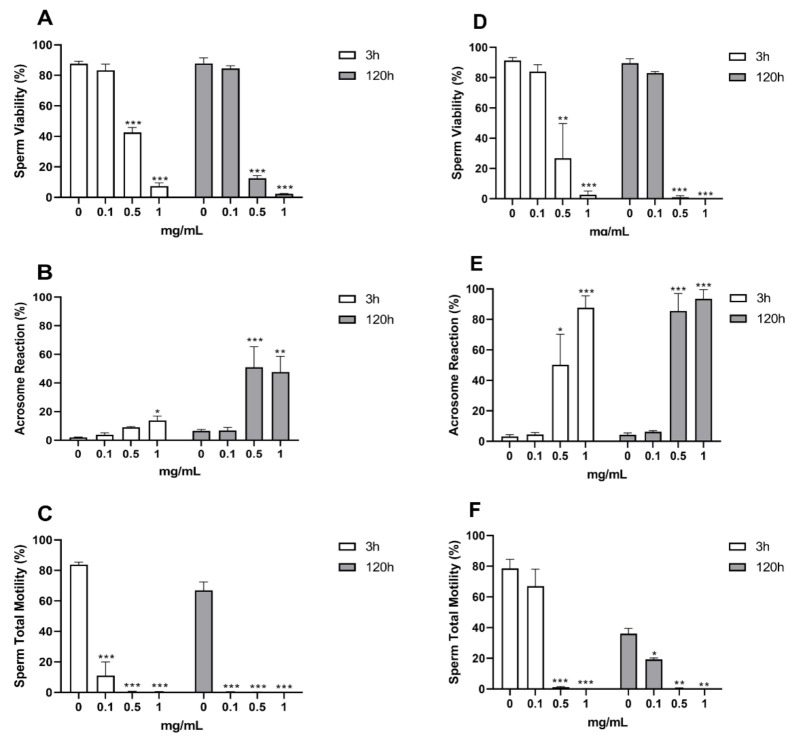
Effects of Cz (**A**–**C**) and Ov (**D**–**F**) EOs on sperm viability (**A**,**D**), acrosome reaction (**B**,**E**) and sperm motility (**C**,**F**). Data are expressed as mean ± standard error of the mean. 0 = control samples (only emulsifiers). * *p* < 0.05; ** *p* < 0.01; *** *p* < 0.001.

The results of the sperm morpho-functional evaluations at 3 and 120 h upon treatment with different concentrations of Cz EO are represented in Figure 2. The analysis of variance showed that the different concentrations statistically altered all analyzed parameters at both time points: viability (3 h, *p* < 0.0001; 120 h, *p* < 0.0001), acrosome reaction (3 h, *p* = 0.0156; 120 h, *p* = 0.0077) and total motility (3 h, *p* < 0.0001; 120 h, *p* < 0.0001).

The results of Dunnett’s tests aimed at comparing EO-treated samples to the control one are reported in Figure 2. The viability of spermatozoa treated with Cz EO (Figure 2A) was statically reduced, at both time points, for the concentrations of 0.5 (3 h *p* = 0.0001; 120 h *p* < 0.0001) and 1 mg/mL (3 h *p* < 0.0001; 120 h *p* < 0.0001). As for the acrosome status (Figure 2B), at 3 h, only the sample treated with the highest EO concentration (1 mg/mL) showed a statistical difference when compared to the control (*p* = 0.0105); yet at 120 h, both 0.5 and 1 mg/mL induced statistically relevant alterations (*p* = 0.0138 and *p* = 0.0196 respectively). Cz EO was capable of inducing significant alterations of sperm motility at all tested concentrations (*p* < 0.0001) at both time points (Figure 2C).

The results of the sperm morpho-functional evaluations at 3 and 120 h upon treatment with different concentrations of Ov EO are represented in Figure 2. As for Cz, Ov EO statistically altered all morpho-functional parameters at both time points: viability (3 h *p* = 0.0002; 120 h *p* < 0.0001), acrosome reaction (3 h *p* = 0.030; 120 h *p* < 0.0018) and sperm motility (3 h *p* = 0.0001; 120 h *p* = 0.0022).

The outcome of the *post hoc* Dunnett’s analysis is reported in Figure 2. At both time points, viability (Figure 2D) significantly decreased in samples treated with 0.5 (3 h *p* = 0.0109, 120 h *p* < 0.0001) and 1 mg/mL (3 h *p* = 0.0002, 120 h *p* < 0.0001) of Ov EO. Accordingly, the same concentrations also determined an increase in the percentage of acrosome reaction (Figure 2E) both at 3 (0.5 mg/mL *p* = 0.0420; 1 mg/mL *p* = 0.0028) and 120 h (0.5 mg/mL *p* = 0.0031; 1 mg/mL *p* = 0.0023). As for sperm motility (Figure 2F), the lowest tested concentration of Ov EO (0.1 mg/mL) did not determine any alteration at 3 h but induced a decrease at 120 h (*p* = 0.0170); the other two concentrations (0.5 and 1 mg/mL) induced complete immobilization of spermatozoa already at 3 (*p* = 0.0002 and *p* = 0.0002, respectively) up to 120 h (*p* = 0.0020, *p* = 0.0019, respectively).

### 3.6. Toxicity of EOs in Galleria mellonella Larvae

**Figure 3:**
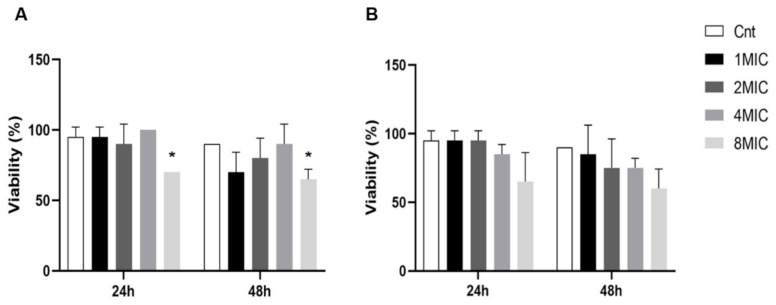
Toxicity of Cz and Ov EOs on *G. mellonella* larvae: (**A**). Plot refers to Cz toxicity; (**B**). Plot refers to Ov toxicity. Data are expressed as mean ± standard error of the mean. * *p* < 0.05 *p*-values were calculated relative to the control.

The results of the *in vivo* toxicity of Cz and Ov EOs on *G. mellonella* larvae are shown in Figure 3. Cz showed no toxicity at 1 MIC, 2 MIC and 4 MIC, while viability was significantly reduced at 8 MIC at 24 and 48 h (*p* = 0.034 for both time points) when compared to the control group larvae (Figure 3A). No toxicity was detected for Ov at any of the tested concentrations (Figure 3B).

## 4. Discussion

Population growth is the main input responsible for increased meat consumption. According to the Agricultural Outlook of the Organization for Economic Co-operation and Development (OECD) and the FAO, global meat consumption will rise by 14% by 2030 [66]. This increasing demand implicated necessary changes in animal production practices and the inevitable introduction of large-scale intensive livestock systems [1]. As a result, the use of antibiotics has considerably intensified, concurring with the actual antibiotic resistance crisis, a serious public health threat of broad concern to countries that compromise drug efficacy, affects multiple sectors and needs to be faced as soon as possible. In 2016, O’Neill estimated that ARBs will cause 10 million deaths per year and a cumulative cost to global productivity of 100 trillion USD by 2050 [67].

As highlighted by European Parliamentary Research Service, the veterinary sector is also responsible for accelerating the spread of antibiotic resistance since farm animals are often reservoirs of pathogens [68,69]. ARBs’ livestock infections are very alarming and have an enormous impact on public health of the potential transfer of resistant strains to humans [70]. In particular, human bacterial zoonosis driven by *Salmonella* spp., *C. perfringens* and *E. coli* can be frequently delivered by contaminated poultry and swine-origin food. For this reason, from 28 January 2022, ambitious rules restricting the use of veterinary antimicrobials started applying across the EU [71].

*Salmonella* spp. colonization in pigs and chickens is a major cause of concern for the veterinary industry. Even if infected animals generally remain asymptomatic carriers, *Salmonella* spp. are responsible for diarrhea, dehydration, fever and death. These infections danger human health as well: it is estimated that *Salmonella* spp. are the cause of over 90 million/year diarrhea-associated diseases in the world, and 85% of these come from food [15]. According to the Center for Disease Control and Prevention (CDC), these bacteria are responsible for about 1.35 million infections in the United States every year [72], while 87,923 confirmed cases of salmonellosis were reported with an EU notification in 2019 [12].

*Clostridium* spp. represent another important problem in livestock. Even if most *Clostridium* spp. are intestinal commensals or inhabitants of the soil, few of them are pathogenic, causing from enteric diseases to neurotoxic and histotoxic ones, both in animals and humans [17]. During 2019, in Europe, 997 of the food-born outbreaks were caused by bacterial toxins, and 82 of these were due to *C. perfringens* and *C. botulinum* [12]. In the USA, *C. perfringens* is one of the most common causes of foodborne illness too, causing nearly 1 million diseases per year [72].

*E. coli* is lately emerging as another global threat. It is generally a widespread commensal, but some strains have acquired virulence-associated genes, empowering them to play an important role as pathogens in humans and animals, causing a wide range of diseases. In humans, pathogenic *E. coli* can cause intestinal illness to urinary tract and life-threatening infections, while in animals, it is mainly associated with diarrhea but can cause edema disease and septicemia too [22,25].

Resistance to the most common antibiotics is increasing in *Salmonella* spp., *Clostridium* spp. and *E. coli* strains isolated from animals and responsible for food poisoning in humans. This limits the treatment options, extends the hospitalization time and complicates the clinical management of severe infections [70,72]. Particularly concerning is the decline in the efficacy of the fluoroquinolones, which are considered among the group of “Critically Important Antimicrobials” according to WHO and which are frequently used in farm animals [70]. The occurrence of resistances to the most commonly used antibiotics has increasingly been documented in *Salmonella* spp. and *Clostridium* spp. strains [73,74,75,76,77]. In particular, *E. coli* strains are developing dramatically high levels of multi-resistances, and especially swine have become extensive reservoirs of ARBs [22,24,78].

Our data underline the current antibiotic resistance concern. Only one *S.* Typhimurium strain was sensitive to all the tested antibiotics, while the other two showed resistance to at least three drugs and reduced sensitivity to both amoxicillin/clavulanic acid association and the cephalosporin cefazolin. All the three *C. perfringens* strains were resistant to lincomycin, with one strain also showing multi-resistances to tetracycline, amoxicillin, doxycycline, erythromycin and penicillin. In this work, *E. coli* strains showed the highest prevalence of multi-resistances, as confirmed by the reported-above literature. One strain was resistant only to tilmicosin and showed reduced sensitivity to ampicillin, while the other three were resistant to at least eight of the tested antibiotics (aminosidine, ampicillin, florfenicol, gentamicin, kanamycin, tilmicosin, sulfamethoxazole/trimethoprim and tetracycline), confirming the alarming role of swine animals in the emergence and dissemination of *E. coli* ARB strains.

In this scenario, natural products are effective tools to reduce ARBs’ spread and diminish the incidence of the main zoonosis in intensive poultry and swine livestock. In particular, EOs are widely studied to fight antibiotic resistance emergence because of their antimicrobial characteristics [42,43,44]. At the same time, EOs provide an alternative to synthetic food additives for which consumers have developed a negative perception [79]. The research of natural compounds with antimicrobial activity is important not only for the direct treatment of infected farm animals but also for prevention purposes. In the past few years, EOs have been supplemented in livestock animals’ diets with a positive effect on growth performances, representing a potential solution for antibiotic-free husbandry nutrition [39,40,80]. EOs have also been nebulized in animals’ houses to enhance hygiene conditions on farms [41].

The EOs considered in the present study exhibited different chemical compositions, especially in terms of monoterpene classes. These differences were reflected in the variable capability in inhibiting the tested bacterial strains. The EOs with the strongest antimicrobial activity were *Origanum vulgare* and *Cinnamomum zeylanicum*, which displayed high concentrations of phenolic monoterpenes, namely carvacrol and eugenol, respectively (Table 2). All the other EOs did not show any trace of phenols in their chemical composition, with the exception of the GR-OLI mixture, where carvacrol represented 35% of the whole composition. On the contrary, the other EOs displayed high concentrations of cyclic hydrocarbons (*Citrus limon*), ethers (*Eucalyptus globulus* and *Melaleuca leucadendron*), cyclic hydrocarbons and alcohols (*Melaleuca alternifolia*) and linear alcohols and derivatives (*Lavandula* and *Mentha piperita*).

Our data show that the most active natural products were the Cz, Ov EOs and mGR-OLI. The IC90 of the Cz (0.13% *v*/*v*) was slightly lower than the Ov (0.25% *v*/*v*) and mGR-OLI (0.33% *v*/*v*) ones. MBCs data reflected those of the MICs, with Cz, Ov and mGR-OLI having the strongest bactericidal activity and showing the same trend as CC90. To evaluate if the volatile compounds of these three best natural products were efficient on *S.* Typhimurium and *E. coli* strains, we performed the micro-atmosphere diffusion assay in which the EOs chemical components were not in direct contact with the bacterial cells. Cz, Ov EOs and mGR-OLI were all capable of significantly inhibiting the bacterial growth, with Cz EO turning out to be the strongest against *S*. Typhimurium strains, while Ov the best against *E. coli* strains. These results confirm the important role played by the EOs vapor phase that had already been highlighted in previous works [81,82,83,84], suggesting the possibility of nebulizing Cz, Ov EOs and mGR-OLI in animals’ houses to reduce bacterial contamination as alternative prophylactic natural treatment able to sanitize the environment of intensive farming.

The *Cinnamomum* genus of the *Lauraceae* family comprises more than 250 evergreen species, and Cz is one of the most studied [85]. Because of its antimicrobial and antioxidant effects, Cz has been applied in pharmaceutical, alternative medicine, cosmetics and food for its flavor and aroma features. Cz EO has already been used as a potential feed additive alternative to antibiotics in poultry, with promising results [86].

The antimicrobial and antifungal activity of Cz EO has been demonstrated, and it is mediated by its principal compounds, cinnamaldehyde and eugenol [87,88,89,90], natural molecules Generally Recognized As Safe (GRAS) by the Flavoring Extract Manufacturers’ Association (FEMA) [91].

In particular, eugenol (generally the major component of *C. zeylanicum* EO obtained from leaves) is an aromatic phenolic compound, which has displayed antimicrobial effects against a wide group of fungi [92,93] and bacteria [94,95,96]. In fungi, eugenol disturbs cell membrane function, inhibits virulence factors and prevents fungal biofilm formation [97]; in bacteria, different mechanisms have been described to explain its activity. Primarily, it could act by disrupting the cytoplasmatic membrane, which increases membrane nonspecific permeability and affects the transport of ions and ATP. Eugenol seems to also be able to induce cell cytotoxicity by producing intracellular reactive oxygen species (ROS), which induces cell growth inhibition, cell membrane disruption and DNA damage, resulting in cell decomposition and death [97,98].

*Origanum* is a genus of herbaceous plants of the *Lamiaceae* family, containing a number of species widely used for culinary purposes and as medicinal plants. *O. vulgare* is the most common, and its EO is currently authorized as a feed additive according to the entry in the European Union Register of Feed Additives (European Commission Regulation No 1831/2003); it can also be used as a flavoring additive in all animal feed, without additional evaluation and approval [12,99]. Migliorini et al. [100] evaluated the effect of Ov EO added to the feed of commercial laying hens, recording beneficial effects on bird health without side effects.

Ov EO has a documented antimicrobial activity against a wide range of microorganisms, such as fungi and bacteria [101,102]. The principal chemical compounds generally found in Ov EO, carvacrol and thymol, are the main mediators of antimicrobial activity [103]. In particular, carvacrol alters the hyphal morphology of fungi, reducing the hyphal diameter and inducing oxidative stress until cell lysis [104,105]; in bacteria, it targets the phospholipid bilayer of cell membranes causing structural and functional damage and increasing the bacterial membrane permeability [104].

Although the antimicrobial activity of eugenol and carvacrol is well documented, these phenols could cause irritation, allergy, and, therefore, exert toxicity at certain concentrations [97,106,107].

mGR-OLI is a modified version of the GR-OLI, an Italian water-soluble mixture of EOs emulsified in an inactive carrier additive, regularly authorized for use in animal feed as an additive. GR-OLI’s antimicrobial properties were first described in 2020 by Di Vito et al. [42].

The toxicity of Cz and Ov EOs was evaluated through two different systems: *in vitro* on porcine spermatozoa and *in vivo* on *G. mellonella* larvae.

As previously reported [59,61], swine spermatozoa can provide preliminary information regarding the different biological effects of EOs. In particular, this *in vitro* approach can differentiate between two key damage mechanisms: membrane disruption (as indicated by viability and percentage of reacted acrosomes) and mitochondrial activity impairment by means of membrane depolarization, potentially leading to the loss of motility [108,109].

Looking at the data from the morpho-functional evaluations of treated spermatozoa, the two tested EOs seem to exert different toxicity patterns. At the middle and highest concentrations (0.5 and 1 mg/mL), both EOs affected all parameters at both experimental points; nonetheless, Cz EO seemed to induce stronger and faster effects on sperm motility and less effects on the membranes, especially when looking at acrosomes. On the other hand, Ov EO showed similar effects, in terms of intensity, for all parameters already at 3 h.

Regarding the lowest tested concentrations (0.1 mg/mL), Cz OE did not show any effect on membranes (cytoplasmatic and acrosomal) but immediately and strongly affected motility. The same concentration of Ov EO did not seem to induce alterations in sperm parameters, with the exception of a mild reduction in motility after 120 h. Overall, Ov EO was well tolerated at 0.1 mg/mL but equally affected all the tested parameters at higher concentrations, while Cz was capable of impairing motility already at the lowest concentration despite membrane disruption being only induced by higher ones. Such different behavior between EOs regarding sperm motility may be imputable to the high content in Eugenol of Cz (>70%); indeed, this compound is capable of altering mitochondrial membrane potential by ATP depletion [110].

In the last years, *G. mellonella* larvae have been used as a model for assessing the *in vivo* efficacy and toxicity of antimicrobial compounds. Larvae have the advantage of being inexpensive to purchase and breed with a short life span, are easy to handle, do not require ethical approval and provide a fast and convenientmean, showing results with a strong correlation to those from mammalian model systems [111,112,113]. Only a few published articles aimed at evaluating the *in vivo* toxicity of natural compounds in *G. mellonella* larvae, and, before this, no study has ever been conducted on Ov EO.

The data showed the toxicity for Cz EO only at the highest concentration tested (8 MIC, i.e., 2% *v*/*v*), while for Ov EO, we detected no toxicity, indicating that this EO was reasonably safe because larvae viability was not significantly altered 48 h after treatment.

In light of their particular features, both spermatozoa and *G. mellonella* larvae proved to be good candidates for toxicological screenings of natural products. Due to the diversity of the two systems, the data showed differences between the *in vitro* and the *in vivo* toxicity, as expected. Indeed, spermatozoa are an extremely sensitive unicellular model, being an organism’s reproductive cells, with toxicity responses necessarily different from those obtained in an *in vivo* study on a multicellular organism such as *G. mellonella* larvae. Therefore, in order to reduce and prevent intensive livestock infections, both EOs can be used alone or in a blend as long as at different concentrations, taking into account these toxicity data.

## 5. Conclusions

In conclusion, our results show that, among all the natural products tested, Cz, Ov EOs and mGR-OLI are the most effective at inhibiting *S*. Typhimurium, *C. perfringens* and *E. coli* strains isolated from poultry and swine. Even if there are several studies on the antimicrobial activity of EOs, there is a scarcity of articles aimed at assessing the antimicrobial activity of EOs’ volatile compounds on strains isolated from animals, and no one has associated *in vitro* and *in vivo* toxicity data before. The study highlights the importance of evaluating natural products to strengthen the hypothesis of safe EO applications for reducing bacterial contamination as effective antibiotic alternatives in complementary therapy. Furthermore, EOs are interesting for preventing farm animals’ infections thanks to their active volatile compounds through the nebulization in animals’ houses, sanitizing confined environments and improving the hygiene standards of intensive livestock and food safety at the same time.

## Figures and Tables

**Table 1:** Bacterial strains used in this study.

D ^1^	Species	Strains	Origin	Year
ST1	*S.* Tiphymurium	343104/3	Chicken	2017
ST2	*S.* Tiphymurium	19173	Pigeon	2018
ST3	*S.* Tiphymurium	344349	Quail	2020
Cp4	*C. perfringens*	318422	Chicken	2021
Cp5	*C. perfringens*	107318	Chicken	2021
Cp6	*C. perfringens*	320897	Chicken	2021
Ec7	*E. coli*	135169	Swine	2021
Ec8	*E. coli*	421464	Swine	2020
Ec9	*E. coli*	140412	Swine	2021
Ec10	*E. coli*	124723	Swine	2021

Note: D ^1^: Designation.

**Table 2:** Relative percentages of abundance of the most representative mono and sesquiterpenes of the essential oils. The whole chemical composition is displayed in Table S1 of the Supplementary Materials.

Components	*LRI* ^1^	*L. × intermedia*	*L. angustifolia*	*M. alternifolia*	*E. globus*	*M. leucadendron*	*O. vulgare*	*C. zeylanicum*	*C. limon*	*M. piperita*	GR-OLI
α-pinene	933	0.40	0.20	4.71	2.91	3.61	0.98	0.09	2.09	0.90	0.92
β-pinene	976	0.39	0.11	0.03	0.50	0.36	0.15	0.02	14.82	1.42	1.17
myrcene	992	0.93	1.06	0.71	0.68	0.39	1.51	-	1.53	0.16	0.58
α-terpinene	1015	0.14	-	9.73	0.18	0.24	1.06	-	0.25	-	0.81
p-cymene	1024	0.65	0.17	3.97	-	1.28	7.04	1.64	0.18	0.18	4.52
limonene	1029	-	0.82	1.85	-	-	0.47	0.26	67.8	2.57	8.89
1,8 cineole	1032	4.74	0.65	2.20	91.44	77.30	-	0.22	-	6.47	6.95
cis-ocimene	1039	0.83	8.51	-	-	-	-	-	-	-	0.38
trans-ocimene	1049	0.50	1.22	-	0.03	-	0.05	-	0.13	-	0.23
γ-terpinene	1059	0.11	0.17	20.64	2.06	0.96	5.50	-	8.61	0.05	4.10
terpinolene	1090	-	-	3.14	-	-	0.15	-	0.35	-	0.39
linalool	1109	35.16	39.69	0.39	-	1.94	1.48	4.27	0.08	0.13	3.23
α-fenchol	1116	0.26	1.13	-	-	-	-	0.12	-	-	-
camphor	1147	6.82	0.20	-	-	-	-	-	-	-	-
menthone	1160	-	-	-	-	-	-	-	0.03	28.07	-
borneol	1169	3.03	0.86	-	-	0.45	0.19	-	-	-	0.41
isomenthone	1169	-	-	-	-	-	-	-	-	9.56	-
terpinen-4-ol	1181	3.48	3.95	40.77	0.05	2.93	0.53	0.26	0.08	-	3.98
menthol	1183	-	-	-	-	-	-	-	-	36.00	-
p-cymen-8-ol	1185	-	0.09	-	-	5.56	-	-	-	-	-
α-terpineol	1193	0.87	1.5	4.40	-	-	-	0.31	0.14	0.44	2.07
myrtenal	1195	0.39	-	1.37	-	-	-	-	-	-	-
geraniol	1261	-	-	-	-	-	-	0.28	-	-	2.28
linalyl acetate	1268	27.97	26.39	-	-	-	-	-	-	-	-
geranial	1275	-	-	-	-	-	-	-	1.19	-	0.17
trans-cinnamaldehyde	1277	-	-	-	-	-	-	-	-	-	14.96
eugenol	1287	-	-	-	-	-	-	70.43	-	-	-
lavandulyl acetate	1295	2.22	2.43	-	-	-	-	-	-	-	-
thymol	1296	-	-	-	-	-	2.78	-	-	-	3.47
menthyl acetate	1298	-	-	-	-	-	-	-	-	4.8	-
carvacrol	1308	-	-	-	-	-	66.98	0.49	-	-	35.61
citronellyl acetate	1358	-	-	-	-	-	-	-	-	-	1.14
neryl acetate	1369	0.28	0.52	-	-	-	-	5.84	0.27	-	-
β-caryophyllene	1426	1.72	2.10	0.03	-	0.47	1.64	4.83	0.14	2.95	2.33
aromadendrene	1446	-	-	1.20	-	-	-	-	-	-	-
β-farnesene	1462	1.24	1.36	-	-	-	-	-	-	-	0.33

Note: *LRI* ^1^: Linear Retention Index.

**Table 3:** Sensitivity of strains to antibiotics.

D ^1^	NA	AN	AMC	AMP	APR	KZ	CT	ENR	FFC	CN	K	TIL	SXT	TE	AX	B	DXT	E	MY	PV	SP	T	TY
ST1	S	S	S	S	S	S	S	S	S	S	S	S	S	S	-	-	-	-	-	-	-	-	-
ST2	S	S	I	R	S	I	S	S	S	R	S	R	S	R	-	-	-	-	-	-	-	-	-
ST3	S	S	I	R	S	I	S	S	S	S	S	R	R	S	-	-	-	-	-	-	-	-	-
Cp4	-	-	-	-	-	-	-	-	-	-	-	-	-	S	S	S	S	S	R	S	S	S	S
Cp5	-	-	-	-	-	-	-	-	-	-	-	-	-	R	R	S	R	R	R	R	S	S	S
Cp6	-	-	-	-	-	-	-	-	-	-	-	-	-	S	S	S	S	S	R	S	S	S	S
Ec7	S	R	S	R	S	S	S	S	R	R	R	R	R	R	-	-	-	-	-	-	-	-	-
Ec8	R	R	R	R	S	R	S	R	R	R	R	R	R	R	-	-	-	-	-	-	-	-	-
Ec9	S	R	R	R	R	R	R	S	R	R	R	R	R	R	-	-	-	-	-	-	-	-	-
Ec10	S	S	S	I	S	S	S	S	S	S	S	R	S	S	-	-	-	-	-	-	-	-	-

Note: D ^1^: Designation; NA: Nalidixic Acid; AN: Aminosidine; AMC: Amoxicilline/Clavulonic Acid association; AMP: Ampicillin; APR: Apramycin; KZ: Cefazolin; CT: Colistin; ENR: Enrofloxacin; FFC: Florfenicol; CN: Gentamicin; K: Kanamycin; TIL: Tilmicosin; SXT: Sulfamethoxazole/Trimethoprim association; TE: Tetracycline; AX: Amoxicillin; B: Bacitracin; DXT: Doxycycline; E: Erythromycin; MY: Lincomycin; PV: Penicillin; SP: Spiramycin; T: Tiamulin; TY: Tylosin; S: Sensitivity; I: Increased exposure sensitivity; R: Resistance.

**Table 4:** Susceptibility testing against natural products: MIC (% *v*/*v*).

	% *v*/*v*									
D ^1^	*E. globus*	*O. vulgare*	*M. alternifolia*	*L. angustifolia*	*M. leucadendron*	*C. limon*	*C. zeylanicum*	*L. × hybrida*	*M. piperita*	mGR-OLI
ST1	1.33 ± 0.58	0.06 ± 0	0.42 ± 0.14	0.5 ± 0	1 ± 0	2 ± 0	0.06 ± 0	0.5 ± 0	0.25 ± 0	0.08 ± 0.04
ST2	1 ± 0	0.13 ± 0	0.67 ± 0.29	1.67 ± 0.58	1 ± 0	>2 ± 0	0.10 ± 0.04	>2 ± 1.53	>2 ± 0	0.25 ± 0
ST3	1 ± 0	0.10 ± 0.04	0.58 ± 0.38	1.17 ± 0.76	1 ± 0	>2 ± 0	0.10 ± 0.04	1.17 ± 0.76	>2 ± 1.88	0.15 ± 0.10
Cp4	2 ± 0	0.25 ± 0	0.83 ± 0.29	1.17 ± 0.76	2 ± 0	>2 ± 0	0.05 ± 0.02	1.17 ± 0.76	1.17 ± 0.76	0.33 ± 0.14
Cp5	1.25 ± 1.06	0.28 ± 0.31	1.25 ± 1.06	1.25 ± 0.06	>2 ± 0	>2 ± 0	0.03 ± 0	1.5 ± 0.71	1.5 ± 0.71	0.38 ± 0.18
Cp6	>2 ± 0	0.21 ± 0.07	1 ± 0	1 ± 0	2 ± 0	>2 ± 0	0.03 ± 0	1 ± 0	1 ± 0	0.21 ± 0.07
Ec7	0.25 ± 0	0.13 ± 0	0.5 ± 0	0.5 ± 0	0.5 ± 0	1 ± 0	0.13 ± 0	0.5 ± 0	0.5 ± 0	0.13 ± 0
Ec8	1 ± 0	0.13 ± 0	0.5 ± 0	0.5 ± 0	1 ± 0	2 ± 0	0.06 ± 0	0.5 ± 0	0.25 ± 0	0.06 ± 0
Ec9	0.5 ± 0	0.13 ± 0	0.5 ± 0	0.5 ± 0	0.5 ± 0	2 ± 0	0.13 ± 0	0.5 ± 0	0.25 ± 0	0.06 ± 0
Ec10	0.5 ± 0	0.25 ± 0	0.5 ± 0	0.5 ± 0	0.5 ± 0	2 ± 0	0.13 ± 0	0.5 ± 0	0.25 ± 0	0.13 ± 0
**IC90**	**2**	**0.25**	**1**	**1.25**	**2**	**>2**	**0.13**	**1.5**	**>2**	**0.33**

Note: D ^1^: Designation.

**Table 5:** Susceptibility testing against natural products: MBC (% *v*/*v*).

	% *v*/*v*									
D ^1^	*E. globus*	*O. vulgare*	*M. alternifolia*	*L. angustifolia*	*M. leucadendron*	*C. limon*	*C. zeylanicum*	*L. × hybrida*	*M. piperita*	mGR-OLI
ST1	1.67 ± 0.58	0.35 ± 0.25	1 ± 0.87	1 ± 0.87	2 ± 1.73	>2 ± 0	0.42 ± 0.51	0.5 ± 0	0.67 ± 0.29	0.67 ± 0.29
ST2	>2 ± 0	1.17 ± 0.76	>2 ± 2.02	>2 ± 0	>2 ± 1.15	>2 ± 0	>0.5 ± 0.38	>2 ± 0	>2 ± 0	1.17 ± 0.76
ST3	2 ± 0	1 ± 0.87	2 ± 0	1.67 ± 0.58	2 ± 0	>2 ± 0	0.25 ± 0	2 ± 0	>2 ± 0	1 ± 0.87
Cp4	>2 ± 0	0.17 ± 0.07	0.83 ± 0.29	1.83 ± 1.89	>2 ± 1.15	>2 ± 0	0.06 ± 0	1.17 ± 0.76	1.17 ± 0.76	0.33 ± 0.14
Cp5	>2 ± 0	0.31 ± 0.27	2 ± 0	2 ± 0	>2 ± 0	>2 ± 0	0.05 ± 0.02	1.5 ± 0.71	1.5 ± 0.71	0.5 ± 0
Cp6	>2 ± 0	0.17 ± 0.07	1 ± 0	1 ± 0	>2 ± 1.15	>2 ± 0	0.03 ± 0	1 ± 0	1.67 ± 0.58	0.17 ± 0.07
Ec7	0.5 ± 0	0.25 ± 0	1 ± 0	1 ± 0	1 ± 0	2 ± 0	0.5 ± 0	1 ± 0	0.5 ± 0	0.5 ± 0
Ec8	2 ± 0	0.25 ± 0	2 ± 0	1 ± 0	2 ± 0	>2 ± 0	0.25 ± 0	1 ± 0	0.5 ± 0	0.13 ± 0
Ec9	1 ± 0	0.25 ± 0	1 ± 0	1 ± 0	1 ± 0	>2 ± 0	0.5 ± 0	1 ± 0	0.5 ± 0	0.13 ± 0
Ec10	2 ± 0	0.5 ± 0	1 ± 0	1 ± 0	>2 ± 0	>2 ± 0	0.5 ± 0	1 ± 0	1 ± 0	0.5 ± 0
**CC90**	**>2**	**1**	**2**	**2**	**>2**	**>2**	**0.5**	**2**	**>2**	**0.67**

Note: D ^1^: Designation.

## Supplementary Materials

The following supporting information can be downloaded at: https://www.mdpi.com/article/10.3390/microorganisms10040822/s1, Table S1. Complete chemical composition of essential oils. The results are expressed as relative percentage of abundance of each mono- and sesquiterpene. Table S2. Micro-atmosphere diffusion assay. Table shows mean values and standard deviations of replicates.
