# Supplementary material for: Potential Applications of Essential Oils for Environmental Sanitization and Antimicrobial Treatment of Intensive Livestock Infections
Source: Microorganisms. 2022 Apr 15;10(4):822. doi: 10.3390/microorganisms10040822 (PMC9029798; doi:10.3390/microorganisms10040822)
Supplement: Supplementary file 1 [file microorganisms-10-00822-s001.zip › microorganisms-1678611-supplementary.pdf]

Supporting Information

Potential applications of essential oils for environmental sanitization and antimicrobial treatment of intensive livestock infections

Melinda Mariotti [1*], Giulia Lombardini [1], Silvia Rizzo [1], Donatella Scarafile [2], Monica Modesto [2], Eleonora Truzzi [3], Stefania Benvenuti [3], Alberto Elmi [4], Martina Bertocchi [4], Laura Fiorentini [5], Lorenzo Gambi [5], Maurizio Scozzoli [6], Paola Mattarelli [2].

[1] Dipartimento di Scienze Biotecnologiche di Base, Cliniche Intensivologiche e Perioperatorie, Università Cattolica del Sacro Cuore, Largo A. Gemelli 8, 00168 Rome, Italy; melinda.mariotti@unicatt.it (M.M.); giulia.lombardini@unicatt.it (G.L.); silvia.rizzo01@icatt.it (S.R.)

[2] Dipartimento di Scienze e Tecnologie Agro-Alimentari, Università di Bologna, Viale G. Fanin 42, 40127 Bologna, Italy; donatella.scarafile2@unibo.it (D.S.); monica.modesto@unibo.it (Mo.M.); paola.mattarelli@unibo.it (P.M.)

[3] Dipartimento di Scienze della Vita, Università di Modena e Reggio Emilia, Via G. Campi 103, 41125 Modena, Italy; eleonora.truzzi@unimore.it (E.T.); stefania.benvenuti@unimore.it (S.B.)

[4] Dipartimento di Scienze Mediche Veterinarie, Università di Bologna, Via Tolara di Sopra 50, Ozzano dell'Emilia, 40064 Bologna, Italy; alberto.elmi2@unibo.it (A.E.); martina.bertocchi3@unibo.it (M.B.)

[5] Istituto Zooprofilattico Sperimentale della Lombardia e dell'Emilia Romagna (IZSLER) – Sede Territoriale di Forlì, Via Don Eugenio Servadei 3E/3F, 47122 Forlì, Italy; laura.fiorentini@izsler.it (L.F.); lorenzo.gambi@izsler.it (L.G.)

[6] Società Italiana per la Ricerca sugli Oli Essenziali (SIROE), Viale Regina Elena 299, 00161 Rome, Italy; mscozzoli@gmail.com (M.S.)

* Correspondence: melinda.mariotti@unicatt.it; Tel.: +39-063-015-4218; Fax: +39-063-051-152

List of contents

Table S1

Table S2

Table S1. Complete chemical composition of essential oils. The results are expressed as relative percentage of abundance of each mono- and sesquiterpene.

**Components**	***LRI*[1]**	***L. x intermedia***	***L. angustifolia***	***M. alternifolia***	***E. globus***	***M. leucadendron***	***O. vulgare***	***C. zeylanicum***	***C. limon***	***M. piperita***	**GR-OLI**
α-thujene	927	0.08	0.13	0.03	-	-	0.82	-	0.36	-	0.17
α-pinene	933	0.40	0.20	4.71	2.91	3.61	0.98	0.09	2.09	0.90	0.92
camphene	947	0.28	0.05	-	0.02	0.03	0.13	0.03	0.07	0.12	0.10
benzaldehyde	958	-	-	-	-	-	-	0.16	-	-	-
sabinene	973	0.12	0.05	0.03	-	0.06	-	-	-	0.37	0.09
β-pinene	976	0.39	0.11	0.03	0.50	0.36	0.15	0.02	14.82	1.42	1.17
octen-3-ol	980	0.23	0.34	-	-	-	0.46	-	-	-	-
3-octanone	987	-	0.60	-	-	-	0.22	-	-	-	-
myrcene	992	0.93	1.06	0.71	0.68	0.39	1.51	-	1.53	0.16	0.58
3-octanol	997	-	-	-	-	-	0.05	-	-	0.11	-
α -phellandrene	1005	0.04	0.07	0.18	0.86	0.22	0.18	-	0.08	-	0.09
δ-3-carene	1010	0.07	-	-	-	-	-	-	-	-	-
α -terpinene	1015	0.14	-	9.73	0.18	0.24	1.06	-	0.25	-	0.81
o-cymene	1022	-	-	0.68	-	-	-	-	-	-	-
p-cymene	1024	0.65	0.17	3.97	-	1.28	7.04	1.64	0.18	0.18	4.52
limonene	1029	-	0.82	1.85	-	-	0.47	0.26	67.8	2.57	8.89
1,8 cineole	1032	4.74	0.65	2.20	91.44	77.30	-	0.22	-	6.47	6.95
cis-ocimene	1039	0.83	8.51	-	-	-	-	-	-	-	0.38
trans-ocimene	1049	0.50	1.22	-	0.03	-	0.05	-	0.13	-	0.23
γ-terpinene	1059	0.11	0.17	20.64	2.06	0.96	5.50	-	8.61	0.05	4.10
trans sabinene hydrate	1067	0.11	-	-	-	-	0.18	-	-	0.05	-
cis linalool oxide	1073	0.2	0.09	0.26	-	-	-	-	-	0.03	-
trans linalool oxide	1089	0.34	0.21	0.02	-	0.15	-	0.12	-	0.04	-
terpinolene	1090	-	-	3.14	-	-	0.15	-	0.35	-	0.39
linalool	1109	35.16	39.69	0.39	-	1.94	1.48	4.27	0.08	0.13	3.23
α-fenchol	1116	0.26	1.13	-	-	-	-	0.12	-	-	-
trans-pinocarveol	1139	-	-	0.52	-	-	-	-	-	0.04	-
camphor	1147	6.82	0.20	-	-	-	-	-	-	-	-
isopulegol	1148	-	-	-	-	-	-	-	-	0.12	-
menthone	1160	-	-	-	-	-	-	-	0.03	28.07	-

borneol	1169	3.03	0.86	-	-	0.45	0.19	-	-	-	0.41
isomenthone	1169	-	-	-	-	-	-	-	-	9.56	-
lavandulol	1171	0.87	0.76	-	-	-	-	-	-	-	-
terpinen-4-ol	1181	3.48	3.95	40.77	0.05	2.93	0.53	0.26	0.08	-	3.98
menthol	1183	-	-	-	-	-	-	-	-	36.00	-
p-cymen-8-ol	1185	-	0.09	-	-	5.56	-	-	-	-	-
α -terpineol	1193	0.87	1.5	4.40	-	-	-	0.31	0.14	0.44	2.07
myrtenal	1195	0.39	-	1.37	-	-	-	-	-	-	-
nerol	1221	-	-	-	-	-	-	-	-	-	-
carveol	1232	0.03	-	-	-	-	-	-	-	-	-
citronellol	1233	-	-	-	-	-	-	0.27	0.04	-	-
pulegone	1244	-	-	-	-	-	-	-	0.80	0.90	-
carvone	1249	-	-	-	-	-	-	-	-	0.07	-
piperitone	1259	-	-	-	-	-	-	-	-	0.65	-
geraniol	1261	-	-	-	-	-	-	0.28	-	-	2.28
linalyl acetate	1268	27.97	26.39	-	-	-	-	-	-	-	-
geranial	1275	-	-	-	-	-	-	-	1.19	-	0.17
trans-cinnamaldehyde	1277	-	-	-	-	-	-	-	-	-	14.96
eugenol	1287	-	-	-	-	-	-	70.43	-	-	-
lavandulyl acetate	1295	2.22	2.43	-	-	-	-	-	-	-	-
thymol	1296	-	-	-	-	-	2.78	-	-	-	3.47
menthyl acetate	1298	-	-	-	-	-	-	-	-	4.8	-
geranyl formate	1305	-	-	-	-	0.10	-	-	-	0.05	-
carvacrol	1308	-	-	-	-	-	66.98	0.49	-	-	35.61
δ-elemene	1341	-	-	-	-	-	-	-	-	0.10	-
α-terpinylacetate	1354	-	-	-	-	-	-	-	-	0.05	-
citronellyl acetate	1358	-	-	-	-	-	-	-	-	-	1.14
neryl acetate	1369	0.28	0.52	-	-	-	-	5.84	0.27	-	-
α-copaene	1380	-	-	-	-	-	-	-	-	0.09	-
geranyl acetate	1387	0.54	0.99	-	-	-	-	-	0.14	-	-
β-bourbonene	1389	-	-	-	-	-	-	-	-	0.18	-
α-gurjunene	1415	0.03	-	-	-	-	-	-	-	-	-
β-caryophyllene	1426	1.72	2.10	0.03	-	0.47	1.64	4.83	0.14	2.95	2.33

α-bergamotene	1441	0.19	-	-	-	-	-	-	-	-	-
aromadendrene	1446	-	-	1.20	-	-	-	-	-	-	-
trans-cinnamyl acetate	1449	-	-	-	-	-	-	-	-	-	0.19
α-humulene	1460	-	-	-	-	-	-	0.11	0.03	0.32	-
β-farnesene	1462	1.24	1.36	-	-	-	-	-	-	-	0.33
allo-aromadendrene	1468	-	-	0.15	-	-	-	-	-	-	-
germacrene D	1488	0.55	-	-	-	-	-	-	-	0.08	-
α-selinene	1500	-	-	-	-	-	-	-	0.03	-	-
viridiflorene	1501	-	-	-	-	-	-	-	-	0.14	-
α-muurolene	1504	-	-	-	-	-	-	-	0.03	-	-
γ-cadinene	1522	0.22	0.05	-	-	-	-	-	-	-	-
δ-cadinene	1530	0.16	-	0.02	-	-	-	-	-	0.04	-
eugenyl acetate	1535	-	-	-	-	-	-	0.89	-	-	-
caryophyllene oxide	1593	0.12	0.29	0.62	-	-	0.14	0.21	-	0.28	0.10
viridiflorolo	1602	-	-	-	-	-	-	-	-	-	-
total		96.31	96.76	97.82	98.73	96.05	92.69	91.03	99.27	97.53	99.47

Note: LRI[1]: Linear Retention Index.

Table S2. Micro-atmosphere diffusion assay. Table shows mean values and standard deviations of replicates.

			cm		
**D[1]**	***C. zeylanicum***	***O. vulgare***	**GR-OLI Mix**	***C. limon***	**Control**
**ST1**	3.93 ± 0.096	2.73 ± 0.15	2.18 ± 0.28	0 ± 0	0 ± 0
**ST2**	3 ± 0.52	1.98 ± 0.39	1.3 ± 0.24	0 ± 0	0 ± 0
**ST3**	3.25 ± 0.19	2.53 ± 0.05	1.1 ± 0.08	0 ± 0	0 ± 0
**Ec7**	2.68 ± 0.05	3 ± 0.48	1.43 ± 0.10	0 ± 0	0 ± 0
**Ec8**	2.98 ± 0.05	3.55 ± 0.37	2.08 ± 0.15	0 ± 0	0 ± 0
**Ec9**	2.9 ± 0.18	2.65 ± 0.06	1.6 ± 0.12	0 ± 0	0 ± 0
**Ec10**	2.83 ± 0.17	3.33 ± 0.38	1.55 ± 0.19	0 ± 0	0 ± 0

Note: D[1]: Designation.
